# Directly tailoring photon-electron coupling for sensitive photoconductance

**DOI:** 10.1038/srep22938

**Published:** 2016-03-11

**Authors:** Zhiming Huang, Wei Zhou, Jingguo Huang, Jing Wu, Yanqing Gao, Yue Qu, Junhao Chu

**Affiliations:** 1 National Laboratory for Infrared Physics, Shanghai Institute of Technical Physics, Chinese Academy of Sciences, 500 Yu Tian Road, Shanghai 200083, People’s Republic of China; 2 Key Laboratory of Space Active Opto-Electronics Technology, Shanghai Institute of Technical Physics, Chinese Academy of Sciences, 500 Yu Tian Road, Shanghai 200083, People’s Republic of China

## Abstract

The coupling between photons and electrons is at the heart of many fundamental phenomena in nature. Despite tremendous advances in controlling electrons by photons in engineered energy-band systems, control over their coupling is still widely lacking. Here we demonstrate an unprecedented ability to couple photon-electron interactions in real space, in which the incident electromagnetic wave directly tailors energy bands of solid to generate carriers for sensitive photoconductance. By spatially coherent manipulation of metal-wrapped material system through anti-symmetric electric field of the irradiated electromagnetic wave, electrons in the metals are injected and accumulated in the induced potential well (EIW) produced in the solid. Respective positive and negative electric conductances are easily observed in n-type and p-type semiconductors into which electrons flow down from the two metallic sides under light irradiation. The photoconductivity is further confirmed by sweeping the injected electrons out of the semiconductor before recombination applied by sufficiently strong electric fields. Our work opens up new perspectives for tailoring energy bands of solids and is especially relevant to develop high effective photon detection, spin injection, and energy harvesting in optoelectronics and electronics.

The motion of electrons can be controlled by photons through band-gap engineering to solids^1^. A basic tenet of energy-band tailoring in materials is that the striking photon radiation excites electrons jumping from ground energy levels up to higher levels and generates charge carriers in the conduction and/or valence bands of the materials^2^,^3^,^4^. It raises the number of mobile charge carriers and makes the material’s electrical conductivity increase^5^. Such positive photoconductance is observed in both bulk and low dimensional photoconductors^6^,^7^. On the contrary, an inverse excitation process of radiation becomes much difficult for carriers to come down to lower levels from the ground states, unless provided that such lower energy states subsist aforehand in the solids. The realization of the inverse process is unprecedented but very attractive to achieve lots of fantastic physics phenomena^8^,^9^,^10^,^11^,^12^, such as negative photoconductance^13^, sensitive terahertz detection^14^, effective spin injection^15^, and controllable semiconductor qubits^16^. It is expected to create such dynamic lower energy states by manipulating the energy bands of material system with photons, which requires to tailor the photon-electron coupling. However, it is very challenging to directly control over the carriers in solids by photons^17^,^18^,^19^,^20^,^21^.

In this Letter, we demonstrate the creation of the lower levels in semiconductors via tailoring of photon-electron coupling in real space. By controlling the materials to respond the anti-symmetric electric field of light to produce an electromagnetic induced well (EIW) in metallic wrapped semiconductor with subwavelength gap distance and selecting different type of semiconductors, we observe not only sensitively positive photoconductance in n-type semiconductor, but also anomalous negative photoconductance in p-type semiconductor realized by injection and trapping of electrons in the EIWs. The photoconductive behavior contrasts with the traditional photoconductivity, which is excited in energy space and usually positive. The light that strikes the semiconductor must have enough energy to raise electrons across the band gap or to excite the impurities within the band gap. Our results reflect the spatial change of electrons in real space originated from the induced potential in the material by the electric field of light. The resultant descent of potential levels increases the electron concentration, which makes the conductivity raised in n-type semiconductor, but reduced in p-type semiconductor. The field-driven variation of photoconductivity by the electromagnetic wave represents an intrinsic property of the coherently coupling interaction of light wave and materials, in contrast to the special photoconductivity arising from defects^22^ or spatial separation of charges by the built-in field in some heterojunctions^23^.

To reveal the general conductive properties of direct manipulation of materials with photons, we selected two kinds of narrow gap semiconductors, n-type of Mercury Cadmium Telluride with nominal compositions of Hg_0.245_Cd_0.755_Te (MCT-1) and Hg_0.231_Cd_0.769_Te (MCT-2) and p-type of Antimony Telluride Sb_2_Te_3_ (ST) (see Figure S1 in Supporting Information). The corresponding band gap energy was 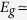 0.23 eV for MCT-1, 0.21 eV for MCT-2, and 0.28 eV for ST at room temperature, respectively. The compositions of MCT were confirmed by their *E*_*g*_, which were determined by the commonly used optical transmission method for MCT. The electron concentration was 7.3 × 10^15^ cm^−3^ for MCT-1, 1.5 × 10^15^ cm^−3^ for MCT-2, and the hole concentration was 7.8 × 10^19^ cm^−3^ for ST. Devices were manufactured by wrapping the two sides of semiconductor with gold films and leave a space gap of *a* = 30 *μ*m for MCT-1 and ST, and *a* = 5 *μ*m for MCT-2 on the top surface of the semiconductor to form a metal-semiconductor-metal (MSM) structure. The devices were investigated by biased current *I*_*b*_ = 1–17 mA under irradiation. In the absence of irradiation, both MCT and ST films exhibit ohmic contact characteristics.

Irradiation with electromagnetic wave of sub-terahertz (sub-THz) frequency alters the conductances through the semiconductor films (Fig. 1). Figure 1(a) shows typical variations in the relative conductance, Δ*σ*/*σ* = (*σ*_*irr*_ − *σ*)/*σ* (*σ*_*irr*_ and *σ* denote conductance in the respective presence and absence of irradiation), recorded for MCT-1 and ST devices periodically exposed to the source of 0.0375 THz frequency in radiation intensity of 10 *μ*W/cm^2^ and 20 *μ*W/cm^2^ under applied bias field *E* of 150 V/cm. A great variation of the conductance occurs, which shows that the photoconductivity is exceedingly sensitive to the irradiation power. When the source is switched on or off, Δ*σ*(*t*) depends on time *t* but not on the intensity of irradiation. It responds timely to the ‘on’ and ‘off’ of 1 KHz modulation frequency. We note that the signs of the observed changes depend on the conduction nature of the semiconductor. When the semiconductor is dominated by electrons, conductance increases on irradiation, Δ*σ* > 0. When, however, the semiconductor is dominated by holes, Δ*σ* < 0.

Figure 1(b) shows the observations related the magnitudes of the relative conductance, Δ*σ*/*σ*, to the power intensity, *P*, of the sub-THz irradiation. For all devices of the two kinds of materials, we find that Δ*σ*/*σ* is linear as a function of the irradiation intensity.

Figure 2 shows the changes related the magnitudes of relative conductance, Δ*σ*/*σ*, to the biased field strength *E* at different modulation frequency for the MCT-1 and ST devices. The change is linearly dependent on *E* below the field intensity of ~180 V/cm for MCT-1. But it deviates the linearity and reaches a maximal variation at ~230 V/cm, then continuously declines up to ~260 V/cm, which is a competitive result between heating and sweeping-out effects of the biased electric field and will be explained later in details. However, the change of ST is linearly on *E* up to >205 V/cm, which means that the heating and sweeping-out effects are unconspicuous in the ST devices.

Both positive and negative sensitivity of the photoconductance can be explained qualitatively by a dynamic model of lower energy well created by the coupling of EM wave to the MSM structure in which the electrons are injected from metals and trapped in the semiconductor. Physically, the MSM structure is as shown in Fig. 3(a,b). For a transverse magnetic (TM) polarized wave, the *x* component of the electric field in the anti-symmetric configuration inside the gap of semiconductor can be written as^24^:


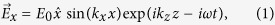


where *E*_0_ is the amplitude of the enhanced EM electric field in the structure, *k*_*x*_ is the wave vector, *ω* = 2*πf* is the angular frequency of light.

The anti-symmetric electric field in *x* direction will produce a corresponding symmetric electrical potential well which can be taken the form *ϕ* = *ϕ*_0_cos(*k*_*x*_*x*)exp(*ik*_*z*_*z* − *iωt*) in terms of *E*_*x*_ = −∇_*x*_*ϕ* − ∂*A*_*x*_/∂*t*, where *ϕ*_0_ is the amplitude of the electrical potential and *A*_*x*_ is vector potential.

According to the Lorentz gauge condition 
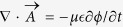
, the *x* component of the electric field can be further expressed as


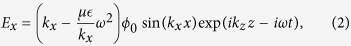


where 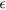 is the permittivity and *μ* is the permeability in free space, and the reciprocal vector is 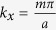 (*m* = 1, 2, ...). In our case, only the fundamental mode needs to be taken into account due to its dominant role, i.e., *m* = 1 here.

When the size of the gap length *a* in the MSM structure is much smaller than the wavelength of incident photons, but much greater than the Thomas-Fermi screening length of electrons^25^, the condition of quasi-static approximation is satisfied and 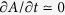 is met in the sub-THz frequency region. Then Eq. (2) can be simplified. Accordingly, the electric field can produce a potential well in real space with the depth distribution as shown in Fig. 3(a) in one half period of the wave oscillations. The electrons in the metals are moved by the external field to inject into the semiconductors, and accumulated in the semiconductor to change the conductance.

Next, in terms of D’Alembert’s equation 
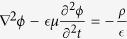
, we find the induced charge density


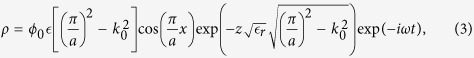


where 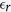 is the relative permittivity of the semiconductor material, and *k*_0_ is the wave vector in free space.

In our structure, the electrons only move forward along *z* direction (Fig. 3(a)) in one half period of the wave oscillations with induced well, but cannot return back in the other half period with induced barrier due to the asymmetric metal-semiconductor structure. So the wave alternations only lead to the injection of extra carriers in the induced well of the semiconductor because of the created lower potential levels.

Integrate the value of *ρ* in *x* and *z* directions, then we can get the averaged variation of the accumulated electron concentration


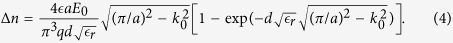


where the electron charge *q* = 1.602 × 10^−19^ C.

The variation Δ*n* increases an electron concentration of Δ*n*_*e*_ in n-type semiconductor, but reduces a hole concentration of Δ*n*_*h*_ in p-type semiconductor. Assumed the variation of the electron and hole mobilities Δ*μ*_*e*_ ≈ 0 and Δ*μ*_*h*_ ≈ 0 when the thermal effect of mobility is negligible, we can derive the output voltage of signal


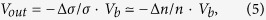


where *n* is the concentration of electrons (or holes) in the n(or p)-type semiconductor before irradiation and *V*_*b*_ is bias voltage on the device.

Eqs. (4) and (5) clearly suggests that external photons can be direclty coupled to electrons in the MSM structure by the wave nature, then produce extra charge carriers Δ*n* in the induced EIW and change conductance of the semiconductor Δ*σ*. It should be noted that only one type of nonequilibrium carriers is generated (electrons in our case), which is different from other ways to generate nonequilibrium carriers, such as light excitation or strong field irradiation, of both electrons and holes.

Figure 3(c–f) depicts the band structures in dark and under irradiation for MCT and ST materials. For n-type material before irradiation (Fig. 3(c)), the Fermi level of MCT is closed to its conduction band and aligned with that of metals. After irradiation, a potential well is formed due to the decrease of the Fermi level of MCT. Electrons from metals inject into the conduction band of the semiconductor (Fig. 3(d)).

However, the Fermi level of ST is closed to its valence band and aligned with that of metals for p-type material before irradiation (Fig. 3(e)). A potential well is also formed under irradiation due to the decrease of the Fermi level of ST. Electrons from metals inject into the valence band of the semiconductor (Fig. 3(f)) in this case.

When the generation rate of electrons is greater than that of recombination rate, the injected electrons can be accumulated either in conduction band for n-type semiconductor or valence band for p-type semiconductor.

To further demonstrate that the signals in Fig. 3 are excited by electromagnetic wave, we carry out frequency response measurements of MCT-1 and ST samples. Figure 4 presents typical frequency-dependent photoresponse of MCT-1 and ST irradiated under fixed sub-THz power modulated in the frequency from 100 Hz to 100 KHz. It obviously shows that the response signal decreases when the frequency increases. The signal of ST decreases faster than that of MCT. We use the formula 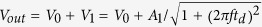 to fit the experimental response curves, where *t*_*d*_ denotes a characteristic decay time constant.

The insets of Fig. 4(a,b) show the fitted *t*_*d*_ for MCT-1 and ST at different biased *E* corresponding to *I*_*b*_ = 1–17 mA, respectively. It demonstrates that the photoconductive response is fast with the order of microseconds. Both *t*_*d*_ decrease when the biased electric field increases. *t*_*d*_ can be decomposed into two parts: recombination time *t*_*r*_, and sweeping time *t*_*s*_, and can be expressed as follows,


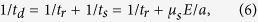


By fitting the corresponding *t*_*d*_ in the two insets in Fig. 4 individually, we obtain *t*_*r*_ and sweeping mobility *μ*_*s*_ for MCT-1 and ST, respectively.

The time constant is *t*_*r*_ = 118.1 *μ*s and *μ*_*s*_ = 0.036 cm^2^/V ⋅ s for MCT-1, and the time constant is *t*_*r*_ = 272.4 *μ*s and *μ*_*s*_ = 0.011 cm^2^/V ⋅ s for ST. The sweeping-out effect impedes the incoming progress of carrier injection, which certainly causes the very small sweeping mobility *μ*_*s*_. It is not difficult to find that the sweeping mobility of MCT-1 is indeed greater than that of ST, which is attributed to a relative smaller scattering rate at lower carrier concentration in MCT-1.

We suggest that the recombination of the accumulated electrons is attributed to electron-hole transition between conduction band and valence band assisted by phonons to transfer the released energy. In narrow gap semiconductors, the recombination rate can be approximately simplified to be ∝exp(*E*_*g*_/2*k*_*B*_*T*) at room temperature^26^,^27^, where *k*_*B*_ is Boltzmann constant, *T* is temperature of material (see Equations in Supporting Information). Substitute *E*_*g*_ of the MCT-1 and ST materials into the above exponential term, the estimated ratio of response speed is ~2.6, which is very consistent with the experimental ratio of ~2.3 calculated from the time constants *t*_*r*_ of the two materials.

Because the conductance of the semiconductors is also sensitive to temperature. Biased electric field *E* will increase the temperature of the semiconductor (see Figure S2 and Figure S3 in Supporting Informtation). We have measured the photoresponse and biased voltage *V*_*b*_ simultaneously at the two ends of the MCT-1 device, then the electron concentration *n* before irradiation and the trapped electron concentration Δ*n* after irradiation of the device can be easily derived in terms of Hall measurement and Eq. (5). Figure 5(a) shows the variation of *n* and Δ*n* of MCT-1 under the source modulation of 1 KHz and 10 KHz frequencies at a large electric field. It shows an increase in *n*, which mainly comes from the enhancement of the MCT temperature due to thermal effect, but a reduce in Δ*n* due to the sweeping of electrons out of the potential well under the large electric field. The sweeping-out effect is to reduce the accumulated carrier concentration Δ*n* in the induced potential well.

Finally, to further investigate the injection and accumulation of electrons produced in the potential well of the semiconductor, a much higher biased electric field than that of MCT-1 is applied to strongly repel the injection of electrons. An MCT material with narrower space gap of *a* = 5 *μ*m is used to achieve a high-intensity field under the same bias currents. Figure 5(b) shows the sweeping-out signals irradiated by the sub-THz source modulated at different frequencies of 1 KHz and 10 KHz for MCT-2. It shows that the signal increases linearly as the biased *E* at a low electric field, but becomes almost saturated when a further increase of the biased *E* is applied. As denoted in Eq. (5), there are three roles for the applied high electric field: to reduce the accumulated carriers Δ*n* by swelling-out effect, to increase the electron concentration *n* because of thermal effect (see Figure S4 in Supporting Information), and to enhance the photovoltage response which is with linear relation to the biased voltage. As a result, they three contribute the *E*-dependent change tendency in conductance. Nevertheless, the decay time constant *t*_*d*_ still decreases even though the photoconductance reaches close to saturation at huge electric field. It means that the injected electrons are swept out with faster speed and more electrons continue to be repelled before recombination at a higher electric field. By fitting *t*_*d*_ curve, we obtain a recombination time constant *t*_*r*_ = 74.1 *μ*s and a slow sweeping mobility *μ*_*s*_ = 1.9 × 10^−3^ cm^2^/V ⋅ s. The *μ*_*s*_ of MCT-2 is much smaller than that of MCT-1. It indicates that a much stronger scattering occurs under high electric field. The estimated drift time of 266.0 *μ*s at the biased electric field of 1000 V/cm, which is greater than the recombination time constant, means that the electrons have sufficient time to recombine before drift to the metals.

In conclusion, we have firstly demonstrated the direct tailoring of photon-electron coupling for extremely sensitive photoconductivity by the injection and accumulation of electrons in the EIWs with excited lower energy levels, which is formed by the interaction of the anti-symmetric electric field of the irradiation with the wrapped MSM structure. Both positive and negative photoconductance have been obviously achieved in n-type and p-type semiconductors, respectively, because the electrons at ground levels in the two metallic sides flow into the lower-level semiconductors. The accumulated electrons may be repelled in reverse process by sweeping-out effect under high biased electric field. Our discovered photoconductance is much different from those reported previously. The result may have great significance to develop novel optoelectronic devices and to facilitate other pending physical processes, such as hole injection in organic semiconductors^28^, spin injection in spintronics^29^, and even qubits adjustments in quantum information.

## Methods

### Material and device manufacture

Mercury Cadmium Telluride (MCT) epitaxial layers of *d* = 8 *μ*m thickness were grown on lattice matched Semi-Insulating substrate Cadmium Zinc Telluride (CZT) by Liquid Phase Epitaxial method. The samples were annealed in the Hg-rich ambient at 240 °C for 24 hours to annihilate the Hg vacancies. The annealed samples were n-type single crystalline semiconductor with electron concentration were 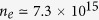 cm^−3^ and 1.5 × 10^15^ cm^−3^, as well as Hall mobilities were 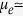 10,000 cm^2^/V ⋅ s and 11,000 cm^2^/V ⋅ s for MCT-1 and MCT-2, respectively. Passivation layers of 100 nm CdTe and 200 nm ZnS were successively grown on the MCT layer to protect the surface of the MCT layer. The ST epitaxial layer of *d* = 2 *μ*m thickness was grown on glass substrate by RF sputtering method at the temperature of 250 °C with hole concentration of 
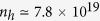
 cm^−3^ and hole mobility 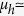 96 cm^2^/V ⋅ s at 300 K by Hall measurement. Devices were manufactured by microelectronic process technology with gold films to wrap the two sides of semiconductor (MCT-1, MCT-2 or ST). Mesas with fixed width of 50 *μ*m were formed by UV photolithography process and mesa etching. It left a space gap of *a* = 30 *μ*m for MCT-1 and ST, and *a* = 5 *μ*m for MCT-2 on the top surface of the semiconductor along *x* axis to form a metal-semiconductor-metal (MSM) structure. Two wires from the end of the two metallic electrodes were connected to a preamplifier, respectively.

## Additional Information

**How to cite this article**: Huang, Z. *et al.* Directly tailoring photon-electron coupling for sensitive photoconductance. *Sci. Rep.* **6**, 22938; doi: 10.1038/srep22938 (2016).

## Supplementary Material

Supplementary Information

## Figures and Tables

**Figure 1:**
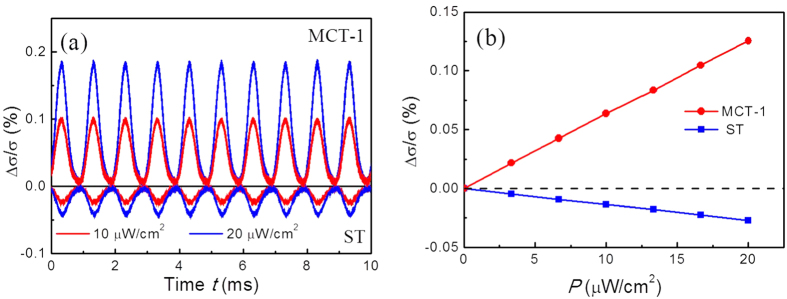
Photoconductance and negative photoconductance of irradiated MSM structure at biased field of 150 V/cm with sub-THz source of 0.0375 THz frequency. (**a**) Change in conductance on irradiation, Δ*σ*/*σ*, for MCT and ST films irradiated periodically at 1 KHz with the intensity of 10 *μ*W/cm^2^ and 20 *μ*W/cm^2^. (**b**) Relative conductance scales linearly with the intensity of irradiating source for MCT-1 and ST.

**Figure 2:**
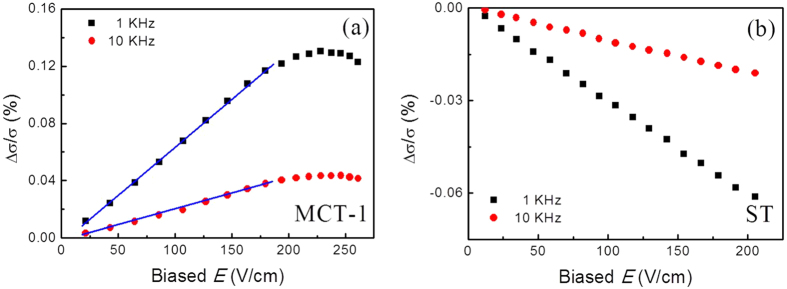
Photoconductance of MSM structure at different biased electric fields irradiated under a given intensity of 10 *μ*W/cm^2^ by sub-THz source of 0.0375 THz frequency. (**a**) Positive change in conductance on irradiation, Δ*σ*, for MCT-1 at the modulated source frequencies of 1 KHz and 10 KHz. (**b**) Negative change in conductance on irradiation, Δ*σ*, for ST at the modulated source frequencies of 1 KHz and 10 KHz. Symbols: Experimental data. Lines: Linear guiding.

**Figure 3:**
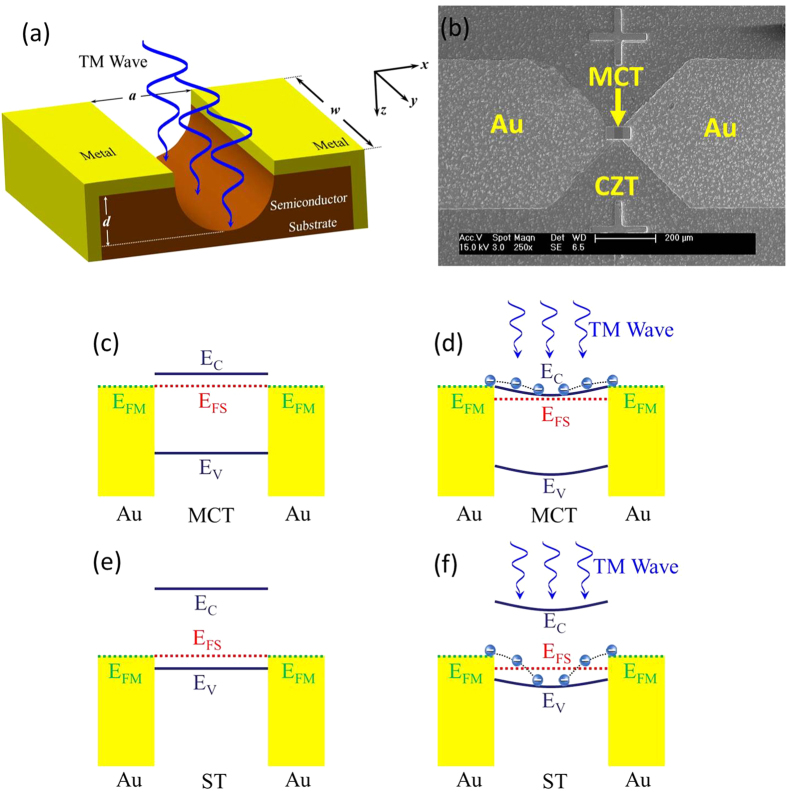
Energy diagrams of MSM structure irradiated by sub-THz source with a gap of length *a* along *x*-axis, width *w* along *y*-axis between two metallic electrodes on the semiconductor with the thickness of *d* along *z*-axis. (**a**) Schematic diagram to form potential well in real space by TM irradiation for electron accumulation in the semiconductor. (**b**) SEM image of MCT-1. (**c**) Band structure of n-type semiconductor with Au-MCT-Au structure before irradiation. (**d**) Band structure of n-type semiconductor with Au-MCT-Au structure under irradiation. (**e**) Band structure of p-type semiconductor with Au-ST-Au structure before irradiation. (**f**) Band structure of p-type semiconductor with Au-ST-Au structure under irradiation. E_C_: conduction band, E_V_: valence band, E_FM_: Fermi level of metals, E_FS_: Fermi level of semiconductors. Blue circles: electrons.

**Figure 4:**
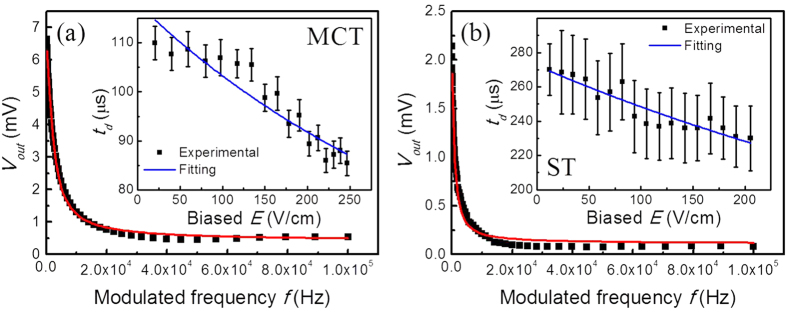
Frequency-dependent photovoltage *V*_*out*_ irradiated by sub-THz source at modulation frequency *f* from 100 Hz to 100 KHz and decay time constants *t*_*d*_. (**a**) *V*_*out*_ and its fitting for MCT-1 biased at 1 mA current. (**b**) *V*_*out*_ and its fitting for ST biased at 1 mA current. Insets: *t*_*d*_ and its fitting as a function of biased field E for MCT-1 and ST, respectively.

**Figure 5:**
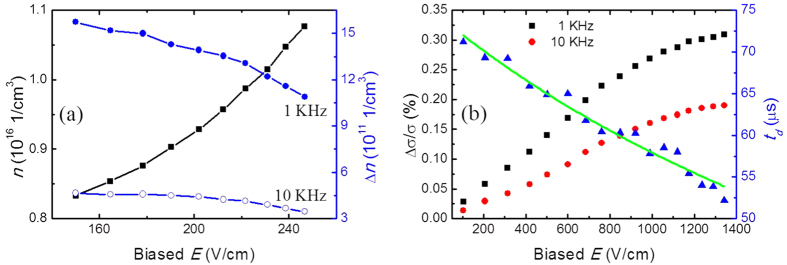
(**a**) Sweeping out of electrons under large biased electric field *E* for MCT-1 irradiated by sub-THz source at modulation frequencies of 1 KHz and 10 KHz. Biased *E* increases the electron concentration *n*, but reduces the injected electron concentration Δ*n* by partially sweeping them out. (**b**) Photoconductance Δ*σ*/*σ* and time constant *t*_*d*_ of the MSM structure for MCT-2 with smaller composition and gap distance at different biased electric field *E* irradiated by sub-THz source. Symbols: experimental data. Green line: theoretical fitting.
