# Supplementary material for: Directly tailoring photon-electron coupling for sensitive photoconductance
Source: Sci Rep. 2016 Mar 11;6:22938. doi: 10.1038/srep22938 (PMC4786808; doi:10.1038/srep22938)
Supplement: Supplementary Information [file srep22938-s1.doc]

Supplementary Information for

“**Directly tailoring photon-electron coupling for sensitive photoconductance**”

Zhiming Huang^1,2^, Wei Zhou^1^, Jingguo Huang^1^, Jing Wu^1^,

Yanqing Gao^1^, Yue Qu^1^, Junhao Chu^1^

^1^National Laboratory for Infrared Physics, Shanghai Institute of Technical Physics, Chinese Academy of Sciences, Shanghai 200083, China. ^2^Key Laboratory of Space Active Opto-Electronics Technology, Shanghai Institute of Technical Physics, Chinese Academy of Sciences, Shanghai 200083, China

1. **Confirming chemical compositions of Mercury Cadmium Telluride**

There are different methods to determine the chemical compositions Mercury Cadmium Telluride (MCT). Among them, optical transmission method is accurate and widely used. Because MCT is transparent below its band gap E_g_, but begins to be [opaque](javascript:void(0);) above E_g_. Here the nominal compositions of MCT are confirmed by their band gaps, which are determined by optical method. Chu et al. [S1] derived the complete formulae of absorption coefficient $\alpha$ of MCT below and above the band gap with different compositions. They showed that $(\ln\alpha)^{2}\propto E-E_{g}$for $E\geq E_{g}$and $(\ln\alpha)^{2}\propto E-E_{0}$for $E<E_{g}$, where $E,\text{ }E_{0}$are photon energy and composition-dependent constant, respectively. Therefore, we can confirm the nominal chemical compositions of MCT-1 and MCT-2 by absorption spectra. Figure S1 shows the absorption coefficient α versus the photon energy $E$ for MCT-1 and MCT-2. The band gap positions E_g_ are determined by the cross points of the two dash lines. They are 0.23 eV and 0.21 eV for MCT-1 and MCT-2, which are corresponding to the nominal compositions of Hg_0.245_Cd_0.755_Te and Hg_0.231_Cd_0.769_Te, respectively.

**Figure S1** (a) Absorption coefficient α versus the photon energy for MCT-1. (b) Absorption coefficient α versus the photon energy for MCT-2. Cross point of the two dash lines based on Chu’s [S1] formulae to determine band gap position E_g_.

1. **Formula of recombination time constant**

The recombination rate of narrow gap semiconductor MCT was shown by Schacham [S2] and Pratt [S3] through the equation

$$R_{r}=n_{i}^{-1}\cdot1.29\times10^{30}(k_{B}T)^{9/2}\exp(-E_{g}/k_{B}T)\text{[}(E_{g}/k_{B}T)^{2}+5E_{g}/k_{B}T+8.75\text{],}$$

where the intrinsic carrier concentration is [S4]

$$n_{i}=(1+3.25k_{B}T/E_{g})\cdot9.56\cdot10^{14}E_{^{g}}^{3/2}T^{3/2}[1+1.9E_{^{g}}^{3/4}\exp(E_{g}/2k_{B}T)\text{]}^{-1}.$$

Consider $E_{g}/k_{B}T>>1$at room temperature in our case, then the recombination rate can be simplified as

$$R_{r}\propto\exp(-E_{g}/2k_{B}T).$$

Therefore, recombination time

$$t_{r}=1/R_{r}\propto\exp(E_{g}/2k_{B}T).$$

It means that the recombination time of different devices can be compared each other based on the band gap, assumed similar properties for different narrow gap semiconductors.

1. **Electron concentration variation**

When a biased voltage is applied to the MSM devices. Joule heating will change the sample temperature. If the heat cannot be transferred immediately from the structure, which will increase the temperature of MCT. Then the electron concentration will be increased. We have measured the biased voltage *V_b_* and current *I_b_* at the two ends of the devices and have estimated the electron concentration at different biased electric fields *E* after accounting for the temperature-dependent mobility in the elevated temperature.

Figure S2 shows the electron concentration *n* as a function of biased electric field *E* for MCT-1 device. The electron concentration only increases slightly before *E*≤107 V/cm, it means that heating and dissipation are almost balanced. However, when *E*>107 V/cm, *n* increases rapidly as the increase of the electric field, which indicates an obvious enhancement for the device temperature. The electron concentration can be adjusted over 30% under electric bias. The Temperature Coefficient of Resistance (TCR) of MCT is ~-1%, it means that the devices has been heated to over 30 degrees above room temperature. In terms of Eq. (5) in the main text, the increase of the carrier concentration will reduce the output of photovoltage if other conditions remain.

**Figure S2** Electron concentration *n* of the device MCT-1 at different biased electric field *E*.

Because the space gap of MCT-2 is much smaller than that of MCT-1, a very high electric field has been achieved in MCT-2 device. Figure S3 shows the variation of electron concentration *n* for MCT-2 device versus the biased electric field *E*. *n* keeps almost constant before *E*≤418 V/cm, but increases fast after that. The change of the electron concentration also increases over 30%. The maximal estimated thermal temperature should be about 30 degrees in terms of the Hall measurement data.

**Figure S3** Electron concentration *n* of the device MCT-2 at different biased electric field *E*.

To further observe the swelling-out effect under higher electric field, we have calculated the concentration variation of the injected carriers. Figure S4 shows the variation of the accumulated electron concentrations Δ*n* irradiated by the sub-terahertz source with the modulation frequencies of 1 KHz and 10 kHz at high biased electric field *E* for MCT-2. It clearly shows that the applied electric field reduces the accumulated carriers Δ*n* by swelling-out effect under high electric field. Furthermore, the increase of *n* also reduces the photovoltage due to the enhanced temperature from thermal effect. Fortunately, the higher biased electric field will promote the photovoltage. As a result, the final *E*-dependent photovoltage is determined by Δ*n*, *n* and biased *E* together.

**Figure S4** Variation of accumulated electron concentration Δ*n* by modulated the source with 1 KHz and 10 KHz frequencies and a comparison to the electron concentration *n* for the device MCT-2 at higher biased electric field *E*.

**Reference:**

S1. Chu, J. H. & Sher, A. Device Physics of Narrow Gap Semiconductors (Springer, New York, 2010).

S2. Schacham, S. E. & Finkman, E. Recombination mechanisms in p-type HgCdTe: Freezeout and background flux effects *J. Appl. Phys.* **57**, 2001 (1985);

S3. Pratt, R. G. *et al.* Minority carrier lifetime in n‐type Bridgman grown Hg_1−x_Cd_x_Te *J. Appl. Phys.* **54**, 5152 (1983).

S4. Chu, J. H., Xu, S. Q. & Tang, D. Y. Energy gap versus alloy composition and temperature in Hg_1−x_Cd_x_Te *Appl. Phys. Lett.* **43**, 1064 (1983).
